# Transurethral enucleation of prostate with button electrode plasmakinetic vaporization for the treatment of Benign Prostatic Hyperplasia

**DOI:** 10.1038/srep39583

**Published:** 2016-12-23

**Authors:** Bo Peng, Jianhua Huang, Guangchun Wang, Haimin Zhang, Min Liu

**Affiliations:** 1 Department of Urology, Shanghai Tenth People’s Hospital, affiliated to Tongji University, Shanghai 200072, China

## Abstract

Benign prostatic hyperplasia (BPH) is a common disease in aged men. In this study, we investigated the efficacy and safety of transurethral enucleation of prostate with button electrode plasmakinetic vaporization for the treatment of BPH. 60 patients diagnosed with BPH who were treated in our hospital from August to December, 2014 by enucleation with button electrode were retrospectively reviewed, and operation time, urinary catheter indwelling time, continuous bladder irrigation time, operation related complications, maximum urinary flow rate (Q_max_), post-void residual urine volume (PVR), International Prostate Symptom Score (IPSS), quality of life assessment (QOL), perioperative hemoglobin and electrolytes were recorded. All the operations were completed successfully. The operation time and urinary catheter indwelling time were 45.3 ± 16.2 min and 1.72 ± 0.32 d, respectively. During the follow-up, urethral stricture (n = 1), and urinary incontinence (n = 2) were found with recovery after 1-month training. Postoperative PVR at 1, 3 and 6 months significantly decreased compared with preoperative ones (*P* < 0.05). IPSS, Q_max_, QOL at 1, 3 and 6 months improved significantly (*P* < 0.05). There was no significant difference in serum hemoglobin, sodium and potassium before and after the operation. Thus, the study proved that enucleation of prostate with button electrode was efficient and safe, which was worth being recommended.

Benign prostatic hyperplasia (BPH) is common in older men, with transurethral resection of prostate (TURP) considered as the gold standard for the treatment of BPH^1^. However, TURP associated complications, such as a relatively high risk of intra- and post-operative hemorrhage, as well as transurethral resection syndrome, still exist^2^. These complications have significantly reduced since the introduction of transurethral plasmakinetic resection of prostate (PKRP), and with its easy and readily accessible features, PKRP is soon applied extensively in clinical practice^3^. Further, PKRP altered the surgical technique from the conventional resection in TURP to enucleation, and from a loop electrode to button electrode, accumulating abundant clinical experience and ideal efficacy. From August to December 2014, 60 patients in our hospital had undergone transurethral enucleation of prostate with button electrode. The results are shown as follows.

## Clinical data

### General information

Sixty male patients aging from 56 to 84 years (mean: 67 years), with a clinical course varying from 1 to 20 years (mean: 7 years) were included in the present study. Medical history included hypertension (n = 32), diabetes mellitus (n = 12), and coronary heart disease (n = 13). Preoperative IPSS, QOL, Q_max_, PVR and estimated prostate weight by abdominal ultrasonography were 19.6 ± 6.8 (14–28), 3.42 ± 1.43, 6.7 ± 3.5 ml/s (ranged 3–12 ml/s), 157.2 ± 87.9 ml (ranged 46–421 ml), 49.22 ± 24.17 g (ranged 17–99 g), respectively.

### Ethics Statement

The operation procedure was performed in accordance with the protocols approved by the clinical Ethics Research Committee, Shanghai Tenth People’s Hospital, Tongji University, following the guideline of urology disease of China.

### Diagnosis and indication

- Age ≥50 years
- Clinically diagnosed BPH patient who fits one or more of these items below were considered as candidate for operation: IPSS ≥8 with poor response to medications; Q_max_ < 10 ml/s; PVR > 60 ml; with recurrent urine retention, prostate hemorrhage and infection; with bladder stone and bladder diverticulum; or with hydronephrosis and secondary renal insufficiency resulting from acute urine retention^4^.

### Operation procedure

Before operation, the informed consents were obtained from all patients. Operation method had been approved by Shanghai Tenth People’s Hospital, Tongji University, and the procedure was carried out in accordance with the guideline of urology disease of China.

The patients were placed in the lithotomy position under general anesthesia with laryngeal mask. Bipolar plasmakinetic resectoscope (Olympus, Japan) with power output set at 280 J for resection and 120 J for coagulation was inserted into the bladder under direct vision. Conventional cystoscopy was performed to check the position of verumontanum, the proliferation of median and lateral lobes, existence of stones or tumors, as well as the condition of urethral orifice and bladder neck, and to estimate the severity of obstruction based on the degree of bladder trabeculation and cellule formation. Incisions were made at 5 and 7 o’clock position of the upper edge of verumontanum, deep into the prostate capsule, and then prostate tissues were vaporized with hemostasis to clear the surgical vision. The median lobe of prostate was enucleated along the surgical capsule by plasmakinetic button electrode. The vessels were coagulated by vaporization from the apex of prostate to the bladder neck and hyperplastic tissues without blood supply were excised by plasmakinetic loop electrode. Two lateral lobes and the tissue at 12 o’clock position around the bladder neck were vaporized and enucleated in proper order under the level of prostate surgical capsule, followed by careful coagulation of any hemorrhagic sources and washing out all the excised tissues. The wound was checked for active hemorrhage and remainder tissue in the bladder after fixing by button electrode. A 22-Fr triple-lumen catheter was inserted into the bladder followed by irrigation until bleeding subsides. Typically, the urethral catheter was removed early on postoperative day 2 or 3 with no obvious bleeding observed in the urine. The procedure obtained the consent of patients.

### Operative parameters

Operation time, perioperative serum sodium and hemoglobin, rate of transfusion, bladder irrigation time, urinary catheter indwelling time and length of hospital stay were recorded. During the 6-month follow-up, postoperative IPSS, Q_max_, PVR and QOL were compared with preoperative ones. Complications including secondary urinary tract infection, hemorrhage, urinary incontinence and urethral stricture were also observed.

### Statistical analysis

Statistical analyses were performed by paired *t* test using SPSS 17.0.

## Results

All the operations were completed successfully. The operation time and urinary catheter indwelling time were 45.3 ± 16.2 min and 1.72 ± 0.32 d, respectively. Bladder irrigation was kept for 12 hours after the operation. During follow-up, urethral stricture was found in one patient, and urinary incontinence was found in 2 with recovery after 1-month rehabilitation training. Postoperative Q_max_ at 1 month, 3 months and 6 months were 26.13 ± 2.27, 27.35 ± 3.42 and 25.43 ± 3.18 ml/s, respectively, and PVR at the same time period were 7.13 ± 3.44, 5.22 ± 2.81, 4.83 ± 2.52 ml, respectively, which were improved significantly (*P* < 0.05, Table 1). Meanwhile, QOL (1.74 ± 0.37, 1.24 ± 0.34, 1.16 ± 35) and IPSS (7.99 ± 2.02, 6.17 ± 2.64*, 5.45 ± 2.01), decreased significantly at postoperative 1, 3 and 6 months, respectively (*P* < 0.05, Table 1). However, there was no statistical difference in hemoglobin, potassium and sodium (*P* > 0.05, Table 2).

## Discussion

TURP has long been the standard treatment for BPH, despite of high incidence of intraoperative and postoperative complications, including bleeding, infection, TUR syndrome, urethral stricture and relapse of BPH. Reich *et al*.^5^ reported that the incidence of complication was as high as 11.1%, which included urinary tract infection (3.6%), massive hemorrhage that require blood transfusion (2.9%), TUR syndrome (1.4%), with 0.1% mortality. In recent research, Komura *et al*.^6^ investigated the 3-year postoperative incidence of urethral stricture to be 6.6%. However, some surgeons, patients, or medias, such as internet, which is very popular currently, have not paid more attention to the complications of TURP, or their information is not accurate^7^.

How to reduce complications of TURP? Perioperative bleeding is one of the most serious complications for surgeons and patients. Kavanagh LE *et al*. reported the Prevention and management of TURP-related hemorrhage, including choice or replacement of anticoagulation and antiplatelet drugs, using of 5-ARIs, and improvement of equipment and process of TURP^8^. Novel techniques have been developed to lower the complication rate, among which bipolar plasmakinetic technology showed promising application in clinics with better function in coagulation, recognition of the capsule, and normal saline as irrigation fluid, thus lowering the incidence of surgical complications. The results also have been reported by Muslumanoglu *et al*.^9^ in their 100-month prospective study. Chinese urologists have been pioneers in applying this new technology by first developing transurethral plasmakinetic enucleation of prostate (PKEP)^10^. PKEP combines the advantages of both transurethral surgery and open enucleation of prostate with the help of plasmakinetic technology instead of resection of prostate as in the traditional practice, and is being recognized by urological specialists worldwide^11^. Further development of the new technique allowed further application of plasma vaporization with use of button electrode. Professor Xie^12^,^13^ first reported favorable clinical outcomes of transurethral vapor enucleation and resection of the prostate with plasma vaporization with button and loop electrode. Compared with PKEP, this technique has several advantages: A. Plasma vaporization with button electrode provides better coagulation due to its large spherical surface. B. It can function as both vaporization and coagulation. C. The capsule is better protected. D. The electrode is less likely to be broken.

The advantages of button electrode includes: A. It can effectively shorten operation time, increase the resection rate of proliferative prostatic tissue, and decrease intraoperative and postoperative hemorrhage for large volume prostate. Chen *et al*.^14^ found PKEP had significant lower rate of hemorrhage and reoperation, and higher rate of resection when compared with transurethral plasmakinetic resection of the prostate (PKRP). Zhang *et al*.^15^ conducted a randomized clinical trial between button electrode and conventional PKRP for large volume prostate (>90 ml) after 3-month follow-up, demonstrating that button electrode had safer and more effective results. In the present research, we verified the efficiency of PKEP with button electrode to have better function in significantly decreasing intraoperative hemorrhage. B. It barely affects the internal environment of patients, providing relative low risk for aged and patients with poor physical condition. Wang *et al*.^16^ carried out a meta-analysis on recent randomized studies on TURP and PKRP, and results showed that the incidence of TUR syndrome was 1–2% and 0% in TURP and PKRP group, respectively. Chen *et al*.^17^ proved great safety and effectiveness of PKRP in aged and high-risk patients. No TUR syndrome and water intoxication were observed since the application of PKEP with button electrode in our hospital, of which the reasons might be the replacement of mannitol by saline, the preservation of the integrity of prostate surgical capsule, as well as the function of occluding vessels so that the water reabsorption is limited. There was no marked difference between preoperative and postoperative serum sodium and potassium in the research, assuring the homeostasis and safety, especially in aged and high-risk patients. C. It can improve the patients’ postoperative quality of life in the short term effectively, which was proved in the report by Zhang *et al*.^18^ that significant increase of postoperative IPSS, QOL and Q_max_ in the group of PKEP with button electrode when compared with conventional TURP group. In our research, the results were the same and patients had better perioperative experience, which corresponds to ‘Comfort Medicine’ being promoted nowadays^19^.

The application of PKEP with button electrode presents favourable short-term outcomes, but with some defects, including capsule perforation and incomplete dissection to treat proliferative and inflammatory tissue in prostate of small volume due to inconspicuous boundary between prostate tissue and surgical capsule. PKEP with button electrode combines the advantages of both transurethral surgery and open enucleation of the prostate, which is worth learning for the doctors equipped with minimally invasive and open surgery technology. For beginners who lack intuitive understanding of prostate open surgery, the learning curve of PKEP is relatively long, but still much shorter when compared with enucleation by holmium laser.

In conclusion, PKEP with button electrode presents promising short-term outcomes for the treatment of BPH with lower perioperative complications, but needs more cases and longer follow-up to confirm long-term efficacy and safety.

## Additional Information

**How to cite this article**: Bo, P. *et al*. Transurethral enucleation of prostate with button electrode plasmakinetic vaporization for the treatment of Benign Prostatic Hyperplasia. *Sci. Rep.* **6**, 39583; doi: 10.1038/srep39583 (2016).

**Publisher's note:** Springer Nature remains neutral with regard to jurisdictional claims in published maps and institutional affiliations.

## Figures and Tables

**Table 1:** Perioperative and Postoperative Q_max_, PVR, IPSS, QOL.

	Preoperative	Postoperative 1 month	Postoperative 3 months	Postoperative 6 months
QMAX(ml/s)	6.70 ± 3.50	26.13 ± 2.27*	27.35 ± 3.42*	25.43 ± 3.18*
PVR (ml)	157.20 ± 87.90	7.13 ± 3.44*	5.22 ± 2.81*	4.83 ± 2.52*
IPSS	19.6 ± 6.8	7.99 ± 2.02*	6.17 ± 2.64*	5.45 ± 2.01*
QOL	3.42 ± 1.43	1.74 ± 0.37*	1.24 ± 0.34*	1.16 ± 0.35*

^*^P < 0.05.

**Table 2:** Perioperative hemoglobin and electrolytes.

	Hemoglobin (g/L)	Potassium (mmol/L)	Sodium (mmol/L)
Preoperative	132.31 ± 9.24	4.21 ± 0.55	142.03 ± 4.68
Postoperative	130.13 ± 12.31	4.17 ± 0.66	141.44 ± 3.97
*t*	1.097	0.3606	0.7447
*p*	>0.05	>0.05	>0.05
